# Mechanisms of the septic heart: From inflammatory response to myocardial edema

**DOI:** 10.1016/j.yjmcc.2024.08.003

**Published:** 2024-08-13

**Authors:** Dihan Fan, Rongxue Wu

**Affiliations:** a Psychiatric Genetics Group, McGill University, Canada; b Department of Medicine, Section of Cariology, Biological Sciences Division, The University of Chicago, IL, United States

**Keywords:** Sepsis-induced cardiomyopathy sepsis, Molecular mechanisms, Endothall barrier function, Cardiac edema, Treatments

## Abstract

Sepsis-induced myocardial dysfunction (SIMD), also known as sepsis-induced cardiomyopathy (SICM), is linked to significantly increased mortality. Despite its clinical importance, effective therapies for SIMD remain elusive, largely due to an incomplete understanding of its pathogenesis. Over the past five decades, research involving both animal models and human studies has highlighted several pathogenic mechanisms of SICM, yet many aspects remain unexplored. Initially thought to be primarily driven by inflammatory cytokines, current research indicates that these alone are insufficient for the development of cardiac dysfunction. Recent studies have brought attention to additional mechanisms, including excessive nitric oxide production, mitochondrial dysfunction, and disturbances in calcium homeostasis, as contributing factors in SICM. Emerging clinical evidence has highlighted the significant role of myocardial edema in the pathogenesis of SICM, particularly its association with cardiac remodeling in septic shock patients. This review synthesizes our current understanding of SIMD/SICM, focusing on myocardial edema’s contribution to cardiac dysfunction and the critical role of the bradykinin receptor B1 (B1R) in altering myocardial microvascular permeability, a potential key player in myocardial edema development during sepsis. Additionally, this review briefly summarizes existing therapeutic strategies and their challenges and explores future research directions. It emphasizes the need for a deeper understanding of SICM to develop more effective treatments.

## 1. Introduction

Sepsis is a life-threatening condition that occurs when the body’s immune system has an extreme response to an infection, causing organ dysfunction [1]. Sepsis accounts for over half of all hospital deaths in the US, making it the primary cause of mortality in hospitals, it is also the leading cause of hospital readmissions and represents the most expensive reason for hospitalization in the US [2–6]. Sepsis-induced cardiomyopathy (SIMD) refers to the impairment of cardiac function resulting from sepsis [7–9], increases the risk of death by up to 70%, and is a major predictor of morbidity and mortality in affected patients [10]. Despite declining age-standardized incidence and mortality rates, sepsis remains a significant global health concern. In 2017, there were 48.9 million cases of sepsis worldwide, resulting in 11 million deaths, which constituted 19.7% of all global deaths [3]. Recent research indicates that sepsis also significantly contributes to mortality in severe COVID-19 cases, with early pandemic data showing a high mortality rate of around 33% for COVID-19-associated sepsis [11,12]. Enhancing sepsis prevention and management strategies is crucial to mitigate its impact on healthcare systems and patient outcomes [9,13].

Despite extensive research on the effect of sepsis on cardiac function, critical care has not sufficiently emphasized this syndrome and effective therapeutic targets remain underdeveloped. Sepsis-induced vascular impairments, such as reduced peripheral resistance and increased microvascular leakage leading to tissue edema, may contribute to myocardial dysfunction [14,15].

The role of myocardial edema (ME) in sepsis-induced cardiomyopathy (SICM) remains poorly studied. ME, the swelling of the heart muscle due to excessive fluid accumulation within the myocardium, is significant in conditions like heart failure and ischemic heart disease [11,16,17]. In sepsis, kinins mediate vascular leakage, causing vasodilation and increased permeability, which result in edema and inflammation [18–20]. Understanding these processes’ impact on cardiac function is critical for managing SICM. This review summarizes the effects of sepsis-induced vascular permeability and ME on cardiac function, providing insights for developing therapeutic targets.

### 1.1. History and definition of sepsis-induced cardiomyopathy (SICM)

Historically, a decreased cardiac index (L/min divided by body surface area in meter square) was used to define myocardial depression since cardiac output can be preserved or even increased in sepsis due to fluid resuscitation and decreased systemic vascular resistance. In 1984, a landmark radionucleotide angiographic study by Parker et al. reported an acute and reversible decrease of left ventricular ejection fraction (LVEF) in septic patients [21], thus describing sepsis-induced cardiomyopathy (SICM) for the first time. During the acute phase of septic shock, the impaired ventricular contractility and ventricular dilatation result in increased end-systolic and end-diastolic volumes, which translates into a decreased ejection fraction (EF) despite preserved or increased cardiac output in these patients. In survivors, this myocardial depression resolves within 7–10 days post-septic shock. Contradictory findings suggest that poor outcome is linked to increased cardiac contractility in reaction to profound vasopelgia, questioning the role of LV dysfunction in increased mortality [22]. Several studies ruled out myocardial ischemia as the cause of LV dysfunction in sepsis by not detecting changes in lactate levels from the coronary sinus flow [23].

SICM occurs as a result of sepsis and is often called septic cardiomyopathy, which is not due to a direct infection of the heart but to the effects of systemic inflammation and the release of cytokines during sepsis [7,24–26]. Despite significant advances over the past four decades, there remains to be a consensus on the definition or criteria for SICM. Nevertheless, SICM is commonly characterized as a reversible dysfunction affecting both systolic and diastolic functions of the left and right heart [27]. It is identified by elevated cardiac biomarkers such as troponins (I/T) and natriuretic peptides (BNP/NT-proBNP), coupled with a clinically observed decrease in cardiac index and left ventricular EF. EF < 50% and a ≥ 10% decrease in the patient’s baseline LVEF, accompanied by an increase in mean end-diastolic and end-systolic volumes (EDV, ESV), often indicates impaired cardiac function [28,29]. Other characteristics of SICM include left ventricular dilatation, normal or low filling pressure, and less response to fluid resuscitation and catecholamines.

EF is the predominant method for assessing left ventricular (LV) function. However, its reliance on loading conditions, particularly afterload, can limit its utility as an indicator of intrinsic myocardial contractility. During SICM, myocardial edema (ME) emerges as a crucial symptom, strongly associated with leakage syndrome. This edema can compress the cardiac chambers, potentially leading to a misleading reduction in LVEF measurements that do not truly represent the myocardium’s inherent contractility. Furthermore, SICM can manifest with preserved EF despite significant cardiac dysfunction attributable to various underlying mechanisms [30]. A recent advancement, speckle-tracking echocardiography (SPE), shows less dependence on loading conditions and may better reflect intrinsic myocardial function. It monitors myocardial movement by tracking ultrasound echoes within the myocardium during the cardiac cycle [31]. Strain, defined as the difference between the resting and final lengths of the myocardium, indicates contractility. Additionally, doppler imaging enhances the sensitivity and specificity of echocardiographic assessment [28,32,33].

Multiple cardiac biomarkers have been studied in sepsis in the past. The use of atrial natriuretic peptides (ANP), brain natriuretic peptides (BNP), C-reactive proteins (CRP), and cardiac troponin I (cTnI) is recognized as a parameter for predicting patient outcomes in sepsis, with BNP identified as the most significant predictor of heart failure and survival among them [34]. Interestingly, increased BNP and ANP can be detected one day before an increase in CRP and cTnI in patients with likely poor outcomes [34], and recent studies have proposed the use of microribonucleic acid (miRNA) as a possible biomarker in sepsis. As miRNAs inhibit protein translation from messenger ribonucleic acids (mRNA), detecting specific sepsis-related miRNA expression changes may help identify poorer outcomes earlier than the well-known protein parameters [35]. Two miRNAs, miR-223 and miR-146, have been identified as potential biomarkers specific to sepsis [35]. Unfortunately, conflicting data from different studies failed to agree upon their prediction value, possibly due to small sample sizes and the insufficient time allowed for their expression [35]. As for SICM, there is no agreement on a specific biomarker for its detection as of now. Thus, identifying a sensitive cardiac biomarker for early diagnosis of SIMD is important for mortality prediction in sepsis.

## 2. Mechanisms proposed for Sepsis-Induced Cardiomyopathy (SICM)

**Fig. 1.**
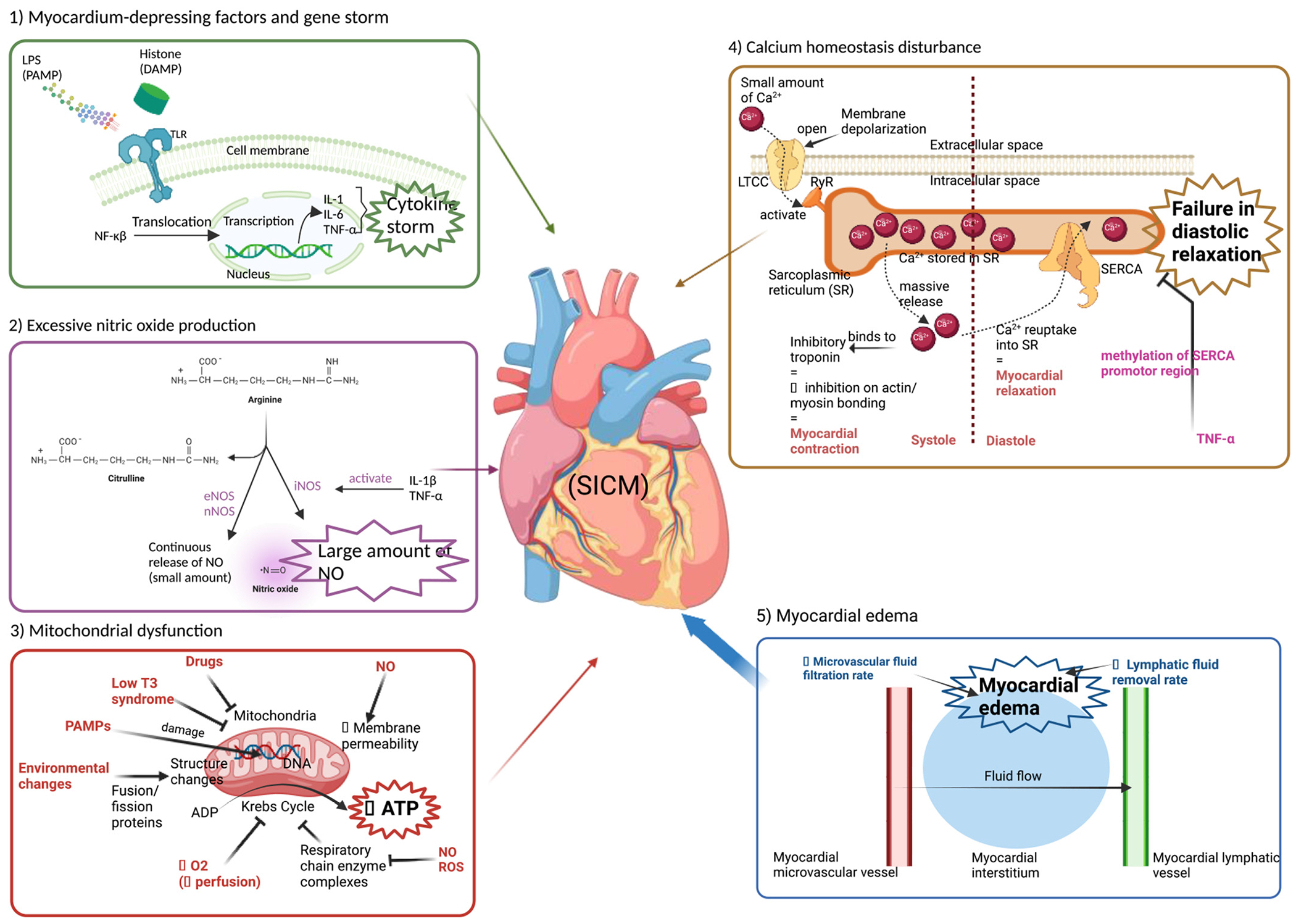
Schematic Overview of Key Pathways in Sepsis-Induced Cardiomyopathy (SICM). *Abbreviations*: LPS = lipopolysaccharide; PAMP = pathogen-associated molecular patterns; DAMP = damage-associated molecular patterns; TLR = toll-like receptor; NF-κB = nuclear factor kappa B; IL-1 = interleukin-1; IL-6 = interleukin-6; TNF-α = tumor necrosis factor alpha; eNOS = endothelial nitric oxide synthase; nNOS = neuronal nitric oxide synthase; iNOS = inducible nitric oxide synthase; IL-1β = interleukin-1β; NO = nitric oxide; T3 = triiodothyronine; ADP = adenosine diphosphate; ATP = adenosine triphosphate; ROS = reactive oxygen species; DNA = deoxyribonucleic acid; LTCC = L-type calcium channels; RyR = ryanodine receptors; SR = sarcoplasmic reticulum; SERCA = sarcoendoplasmic reticulum calcium ATPase; SICM = sepsis-induced cardiomyopathy

The mechanisms behind SICM are not fully understood. Initially, SICM was thought to result from global myocardial ischemia due to inadequate coronary blood flow. However, studies have shown preserved or increased coronary blood flow in septic shock patients with myocardial dysfunction, refuting this hypothesis [36]. Inflammatory cytokines such as IL-1, IL-6, and TNF-α are hypothesized to contribute to SICM. However, clinical trials targeting specific pro-inflammatory cytokines have not shown convincing evidence to improve outcomes. Other factors, such as the complement system and pathogen-associated molecular patterns (PAMP), are also involved. Recently, residual myocardial edema in septic patients following recovery has led to the revaluation of endothelial dysfunction and leakage in SICM pathogenesis [14,15]. Other mechanisms, like oxidative stress, mitochondrial dysfunction [37], and altered calcium homeostasis, are also being studied shown in (Fig. 1).

1. **Myocardium-Depressing Factors and Gene Storm:** Pathogen-associated molecular patterns (PAMPs) like lipopolysaccharide (LPS) and damage-associated molecular patterns (DAMPs) such as histone activate toll-like receptors (TLRs). This activation triggers the translocation of NF-κB into the nucleus, leading to the transcription and production of inflammatory cytokines (IL-1, IL-6, TNF-α), culminating in a ‘gene storm.’
2. **Excessive Nitric Oxide Production:** Inflammatory cytokines (IL-1β, TNF-α) activate inducible nitric oxide synthase (iNOS), resulting in the excessive production of nitric oxide (NO). This exacerbates cardiac dysfunction through sustained NO release, which differs from the small amounts typically produced by endothelial (eNOS) and neuronal (nNOS) nitric oxide synthases.
3. **Mitochondrial Dysfunction:** Various factors such as environmental changes, drugs, and PAMPs cause structural and functional damage to mitochondria. This includes disruptions in ATP production, low T3 syndrome, increased membrane permeability, and oxidative damage (NO and ROS), all contributing to reduced oxygen availability and energy production, leading to cardiomyocyte ‘hibernation.’
4. **Calcium Homeostasis Disturbance**: Inflammatory cytokines interfere with calcium handling in cardiomyocytes. The sarcoplasmic reticulum (SR) releases calcium through ryanodine receptors (RyR) but struggles to reuptake it due to inhibited sarcoendoplasmic reticulum calcium ATPase (SERCA) function. This leads to decreased myocardial relaxation during diastole and impaired contraction during systole, contributing to failure in diastolic relaxation.
5. **Myocardial edema**: Increased microvascular fluid filtration rate and decreased lymphatic fluid removal rate led to fluid accumulation in the myocardial interstitium. This myocardial edema impacts cardiac function by disrupting the balance of fluid flow, exacerbating cardiac dysfunction.

### 2.1. Role of inflammatory cytokines in SICM: initial beliefs and current understanding

The overactivation of the innate immune system at the onset of sepsis releases damage-associated molecular patterns (DAMPs), initiating a cytokine storm characterized by excessive inflammatory cytokines [38]. This leads to widespread cardiac injury, manifesting as reduced cardiac function and structural damage. Myocardium-depressing factors (MDF) are substances responsible for decreased cardiac function during sepsis. In a septic patient, different PAMPs, such as lipopolysaccharide (LPS) present in certain microorganisms, and endogenous DAMPs, such as high mobility group box 1 (HMGB1) and extracellular histones resulting from damaged tissues, are recognized by Toll-like receptors (TLRs), a trans-membrane glycoprotein, classically known to be expressed on the surface of macrophages and dendritic cells (Fig. 1). TLRs are also found on the surface of myocardial cells, which express TLR2, TLR3, TLR4, and TLR9 [39]. Aside from TLR3 [40], all TLRs signal through the myeloid differentiation factor 88 (MyD88)-dependent pathway and activate c-Jun N-terminal kinase (JNK), extracellular signal-regulated kinases 1/2 (ERK1/2), p38 mitogen-activated protein kinase (MAPK) and the transcription factor nuclear factor (NF)-κB signaling pathways. The nuclear translocation of (NF)-κB activates the expression of multiple pro-inflammatory cytokines, a phenomenon called gene storm, which includes the production of IL-1, IL-6, and TNF-α [41]. Additionally, it has been recently shown that exosomes issued from bacterial-infected macrophages contribute to this inflammatory state, and the blockade of macrophage exosome release in septic mice models resulted in decreased circulating pro-inflammatory cytokines, attenuated cardiac depression, and improved survival [42]. In the heart, the expression of pro-inflammatory macrophages (M1) and anti-inflammatory macrophages (M2) is likely modulated by growth differentiation factor 3 (GDF3), which suppresses the former phenotype (M1) when secreted in large quantities in mice models; However, despite the initial anti-inflammatory benefits during the acute systemic response in sepsis, persisting high levels of circulating GDF3 are believed to shift M1 towards the M2 phenotype, consequently immunosuppressing the patient and increasing mortality [43]. Parallelly, circulating plasmatic exosomes in sepsis are mostly released by platelets and are shown to greatly enhance the formation of neutrophil extracellular traps (NETs), which fulfill the bactericidal role in the systemic response to pathogens but also act as potent inflammatory response promoters [44]. Exosomal HMGB1, miR-15b-5p and miR-378a-3p have been identified in septic mice models as the possible exosomal components responsible for NET formation in sepsis, in which the platelet exosome release is likely regulated by IκB kinase (IKK) [44].

Pilot studies have investigated the role of cytokines in the pathogenesis of SICM since the 1990s, as Vincent et al. noticed a transient improvement of ventricular function in six of the ten septic patients following the administration of murine monoclonal anti-TNF antibody (2 mg/kg), defined by the authors as decreased cardiac rate with improved left ventricular stroke work index (LVSWI) and unchanged cardiac filling pressure [45]. However, in the subsequent NORASEPT II double-blind, randomized controlled trial involving 1879 patients, no improvement in survival was seen in patients treated with anti-TNF monoclonal antibody [46]. These results were met with criticism from Dr. Johnathan Cohen for the lack of serum TNF-a concentration data, suggesting possible treatment ineffectiveness in vivo, but also questioning the recruitment criteria in general for septic patients as it covers infections from different pathogens, thus failing to conceive a homogenous study group [47]. Around the same year, in an induced-sepsis animal model where mice injected with anti-TNF-alpha monoclonal antibody prior to intravenous injection of *Escherichia coli* endotoxin were compared to mice who were not injected with anti-TNF antibody, Boillot et al. noticed that anti-TNF antibody did not prevent endotoxin-induced vascular hyporeactivity to norepinephrine, but partially attenuated the decrease in maximal response to norepinephrine [48]. Currently, there is not enough clinical evidence to determine the exact role, if there is any, of inflammatory cytokines in SICM in humans. However, considering their short half-lives, TNF-a and IL-1 are also believed by some authors only to be responsible for the early cardiac depression seen in SICM and the activation of excessive myocardial nitric oxide (NO) synthesis, a much more critical factor for the perpetuation of cardiac depression in SICM [49]. This theory would explain the failure to obtain clinical differences in experiments relying solely on inflammatory cytokines.

### 2.2. The role of excessive Nitric Oxide (NO) production in SICM

**Fig. 2.**
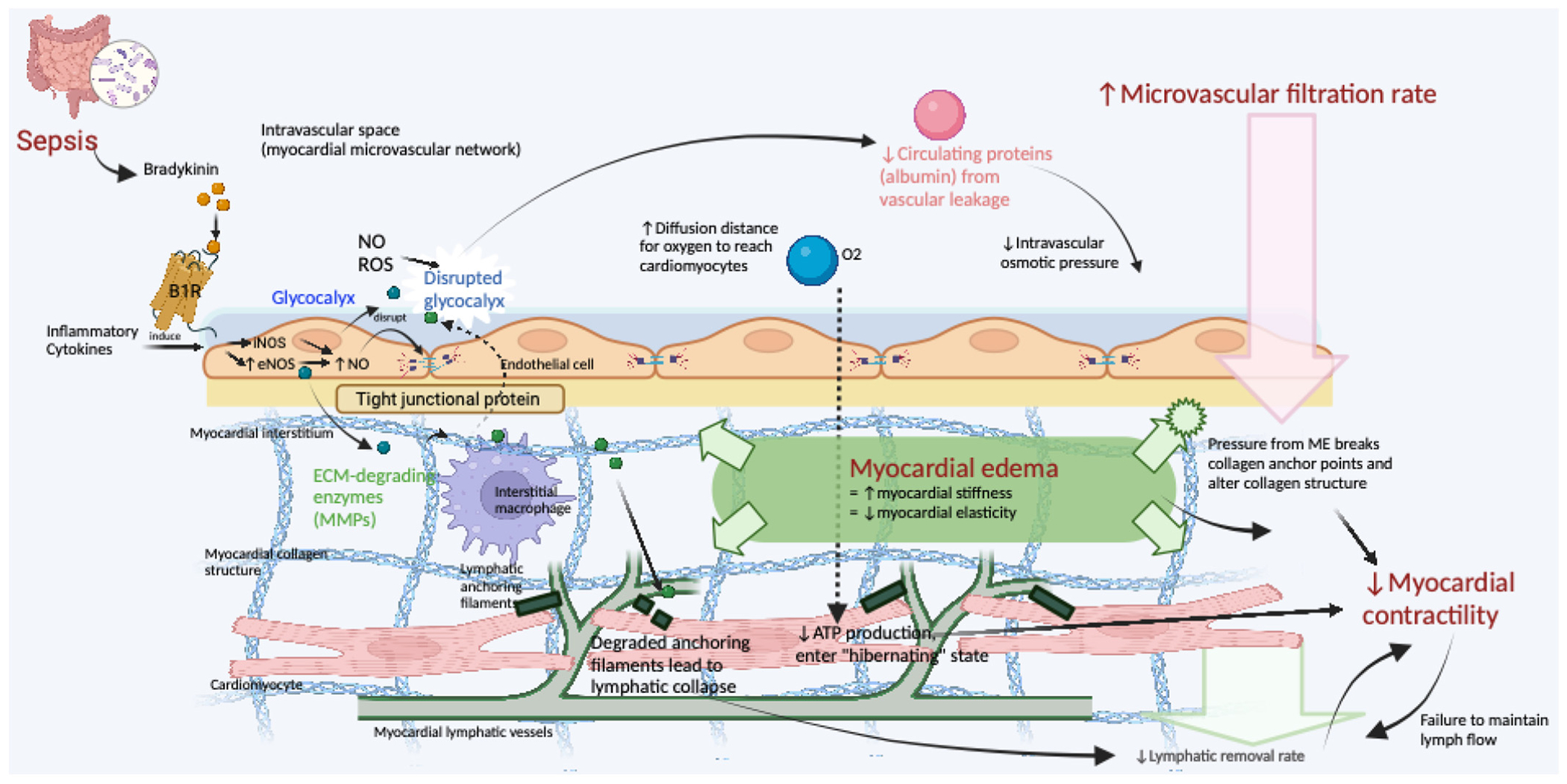
Schematic of Myocardial Edema Development in Sepsis-Induced Cardiomyopathy (SICM). In sepsis, bradykinins, upregulated by inflammatory cytokines, bind to the B1R on endothelial cells, stimulating nitric oxide (NO) production via endothelial nitric oxide synthase (eNOS). This NO surge disrupts endothelial tight junctions and the glycocalyx, increasing vascular permeability. Concurrently, matrix metalloproteinases (MMPs) degrade structural proteins, causing lymphatic vessels to collapse and impeding fluid drainage. The leakage of albumin into the interstitium lowers vascular osmotic pressure, exacerbating fluid filtration into the myocardium. The resulting myocardial edema (ME) compresses myocardial structures, disrupting collagen integrity and increasing stiffness, which diminishes myocardial elasticity and contractility. Moreover, compromised cardiac contractions hinder lymphatic outflow, creating a feedback loop that perpetuates ME and cardiac dysfunction. *Abbreviations*: B1R = bradykinin receptor B1; eNOS = endothelial nitric oxide synthase; NO = nitric oxide; ROS = reactive oxygen species; ECM = extracellular matrix; MMPs = matrix metalloproteinases; ATP = adenosine triphosphate

Nitric oxide (NO) is generated from the oxidation of L-arginine into L-citrulline by the enzyme nitric oxide synthase (NOS) (Fig. 2). In myocardial cells, three isoforms of NOS have been identified: endothelial NOS (eNOS), neuronal NOS (nNOS) and inducible NOS (iNOS). Unlike endothelial and neuronal NOS, which continuously generate a small amount of NO, inducible NOS is only stimulated by inflammatory cytokines (such as IL-1B and TNF-a) and produces a large amount of cytosolic NO upon expression [50]. The activation of eNOS and nNOS, which are considered constitutive NOS, seems to modulate cardiac myocyte responsiveness to muscarinic cholinergic and beta-adrenergic receptor stimulation [51]. The activation of iNOS is necessary but not sufficient by itself to cause a marked depression in myocyte contractile responsiveness to beta-adrenergic agonists [52,53], and is suggested to play a key role in SICM. Many other mechanisms are proposed to explain the contribution of iNOS activation in SICM, including changes in both preload and afterload, a reduction in the response of cardiac myofilaments to Ca^2+^ secondary to NO-induced overexpression of cyclic guanosine monophosphate (cGMP) [54] and NO’s participation in mitochondrial dysfunction. This agrees with a study on septic mice models, in which increased mitochondrial permeability is associated with the overexpression of mitochondrial NOS [55,56]. Recently, the administration of nicotinamide riboside (NR), a precursor of nicotinamide adenine dinucleotide (NAD^+^), which plays an important role in oxidative stress regulation and is decreased in sepsis [57,58] has been shown to improve survival in septic mice models by attenuating pulmonary micro permeability and cardiac dysfunction [59]. This is believed to be the effect of inhibited high mobility group box-1 (HMGB1) release and inhibited oxidative stress via the NAD^+^/sirtuin 1 signaling in the treated mice [59].

### 2.3. Mitochondrial dysfunction as a link to cardiac failure in Sepsis

Proper mitochondrial function is essential for ATP production in the heart. Necessary in all energy-consuming organs, including the heart, as it is the major source of the high-energy compound adenosine triphosphate (ATP). The morphology of mitochondria is dynamically regulated by a balance between fusion and fission processes. This delicate equilibrium is orchestrated by specific proteins: mitofusins 1 and 2, optic atrophy 1 (OPA1) for fusion, and dynamin-related protein 1 (Drp1) and mitochondrial fission protein 1 (Fis1) for fission. These proteins are highly sensitive to changes in the mitochondrial environment, ensuring proper adaptation to cellular demands [60]. During sepsis (Figure 1), mitochondrial dysfunction is caused by alterations in mitochondrial morphology, oxidative damage to mitochondrial DNA by pathogen PAMPs [61], increased permeability from NOS overexpression [62], and downregulated genes transcribing mitochondrial proteins in early inflammatory response [63]. Downregulated genes related to mitochondrial changes are associated with hormonal alterations from the well-recognized ‘low T3’ syndrome in sepsis [64–66]. These changes impair ATP production, further exacerbated by reactive oxygen species (ROS) and nitric oxide (NO) inhibiting the respiratory electron transport chain complexes (notably complex I and IV) [67–69]. Moreover, drugs used in critical care can inhibit mitochondrial function [70].

Energy metabolism plays a critical role in the pathophysiology of sepsis-induced myocardial dysfunction (SIMD). The heart typically relies on oxidative phosphorylation for ATP production, mainly through fatty acid and pyruvate oxidation. However, sepsis induces a metabolic shift that impairs the oxidation of both fatty acids and glucose, leading to reduced ATP production. Increased superoxide anion production within mitochondria and the formation of toxic peroxynitrite further impair mitochondrial function. This metabolic dysfunction contributes to overall cardiac dysfunction in sepsis. Hyperlactatemia, common in sepsis, results not solely from tissue hypoxia but also from adrenergic stimulation and Na+/K + -ATPase activation, increasing energy demand on a failing heart. [71]

As cellular metabolic activity continues with insufficient energy, ATP levels drop, activating cell death pathways, though cell death does not seem to play a major role in SICM [72]. Since non-mitochondrial ATP production through enhanced glycolytic activity is a short-term solution and cannot completely compensate for mitochondrial ATP production [73], cells enter a state of hibernation. In the human heart, hibernation is a well-recognized phenomenon in which cardiomyocytes decrease their activity in response to decreased myocardial perfusion [74], which may explain the depressed cardiac function in SICM. Additionally, sustained mitochondrial dysfunction is also believed to be responsible for the often observed persisting muscle weakness in sepsis survivors, which could not be explained by muscle atrophy alone [75]. A study on the muscles of mice septic model survivors showed persistent mitochondrial structural modifications and functional impairment [75], which may give insight into the pathogenesis of the lasting effects of SICM on cardiac function.

### 2.4. The role of calcium homeostasis disturbance in SICM

The physiological cardiac cell contraction revolves around a Ca2 + −induced Ca2 + −release (CICR) process. During phase 1 of the action potential, membrane depolarization triggers the opening of the voltage-sensitive sarcolemmal L-type Ca^2+^ channels (LTCC), which allows a small amount of Ca^2+^ to enter the cell. This activates the SR Ca^2+^-sensitive release channels, specifically the sarcoplasmic reticulum (SR) ryanodine receptors (RyR) that form structures called “dyads” in juxtaposition to LTCC. Consequently, Ca^2+^ stored in the SR is released, and this transient rise in cytosolic Ca^2+^ activates myofilament contraction by binding to the inhibitory troponin, which removes its inhibitory effect on actin/myosin binding, leading to their cross-linkage [76]. During diastole, cardiac cell relaxation is achieved by 1) Ca^2+^ reuptake from the cytosol into the SR via SR Ca^2+^ ATP-ase (SERCA), which is tightly regulated by its main regulatory subunit phospholamban (PLB), and by 2) extrusion from the cell through the sarcolemmal Na^+^/Ca^2+^ exchange. Consequently, the amount of Ca^2+^ stored in the SR is regulated by SERCA, and inhibition of SERCA leads to the failure in diastolic relaxation as Ca^2+^ reuptake into the SR is blocked (Fig. 1). In SICM, inflammatory cytokines play an important role in Ca^2+^ homeostasis. It has been demonstrated that TNF-alpha increases DNA methyltransferase levels, thus enhancing the methylation in the SERCA promoter region, resulting in reduced SERCA [77], and the prevention of SERCA suppression in murine polymicrobial sepsis models has shown to improve cardiac function and outcome [78]. Interestingly, a recent discovery suggests that increased intracellular calcium in sepsis may also contribute to the over-activation of calpain, an intracellular calcium-dependent protease that is believed to potentialize inflammatory cytokine release and cellular apoptotic pathways [79]. During sepsis, calpain production is likely to be furthermore enhanced by increased NO activities and decreased expression of calpastatin, an endogenous calpain-specific inhibitor [79]. In a transgenic mice model overexpressing mitochondrial-targeted calpastatin in cardiomyocytes, myocardial mitochondrial ROS production was successfully prevented as the mice underwent induced myocardial ischemia/reperfusion, ultimately decreasing cardiomyocyte death, thus reducing myocardial injury from ischemia [80]. These discoveries underline calpain as a possible treatment target for SICM and other oxidative stress-related cardiomyopathies.

### 2.5. The role of Myocardial Edema (ME) in SICM

During SICM, myocardial edema is a critical aspect that complicates cardiac function. ME, defined as excess fluid accumulation in the myocardial interstitium, is the consequence of an imbalance between the microvascular fluid filtration rate and the lymphatic fluid removal rate (Figure 2), with the former being greater than the latter [81]. Despite studies on animal models in the past decades suggesting the role of ME in SICM, it has only been brought back to the spotlight recently following interesting clinical studies such as the case reports by Vasques-Nóvoa et al. [82], which noted the shared presence of residual ME in three septic middle-aged patients with no previously known cardiovascular disease. The first patient, a 55-year-old man admitted to ICU for bacteremia pneumococcal pneumonia and the second patient, a 43-year-old woman admitted to ICU for *Escherichia coli* bacteremia following obstructive pyelonephritis, were both presenting with a mild high-sensitivity cardiac troponin I (hs-cTnI) elevation at the start followed by a marked troponin elevation on day 2–3, as well as coincidental marked left ventricular (LV) diastolic dysfunction (E’ ~ 5 cm/seg) and global systolic dysfunction with severely depressed LV ejection fraction (30%). Following a favorable clinical course with normalized hs-cTnl and gradually improved systolic and diastolic function, cardiac magnetic resonance imaging (CMR) of patients 1 at 28 days and 2 at 49 days suggested residual ME and subepicardial remodeling. Interestingly, scar tissue formation was also noted in patient 1 subsequently despite negative tests for coronary artery disease (CAD) in both patients. Patient 3, a 51-year-old man with unremarkable medical history who presented with bacteremic pneumococcal pneumonia died on day 2 following refractory septic shock. Despite the absence of anatomopathological evidence of CAD or thrombosis, confocal microscopy studies on a post-mortem transmural left ventricle free wall (LVW) sample revealed interstitial edema in every segment of LVW, notably at the subepicardial region. Further marker studies on this sample revealed the frequent association between increased extracellular matrix (ECM) disruption and CD163-positive inflammatory cells (macrophages and monocytes), increased distance between capillaries and cardiomyocytes, as well as focal myocytolysis in edematous areas.

The effects of ME on cardiac function have been studied during the last decade and ME is considered a frequent pathology in response to many clinical states, including arterial hypertension and hypoproteinaemia, as well as clinical interventions such as cardiopulmonary bypass and cardioplegic arrest during cardiothoracic surgeries. It is also found that myocardial edema leads to pulmonary arterial hypertension and pericardial effusion [83]. Unlike other organs, which can tolerate increases in interstitial fluid volume and accumulation or edema formation without a compromise in function, the heart is extremely sensitive to the accumulation of myocardial interstitial fluid, which significantly affects cardiac function even if it increases by a small percentage [84]. Myocardial edema dramatically reduces energetic efficiency, impairing both contraction and relaxation [85].

#### 2.5.1. Capillary Leakage Syndrome (CLS) and the role of plasma colloids in maintaining microvascular permeability

One of the most important factors determining the fluid filtration rate is the myocardial microvascular permeability, which has been reported to significantly increase in both experimental and clinical sepsis as early as in the 1990s [86], along with accompanying decreased cardiac function. Sepsis most commonly causes secondary capillary leakage syndrome (CLS), which significantly contributes to cardiac edema. This phenomenon is characterized by disruption of the endothelial barrier within capillaries, primarily due to the action of inflammatory mediators. During sepsis, cytokines such as TNF-α, IL-1β, and IL-6 increase endothelial permeability [87]. Simultaneously, microbial products like lipopolysaccharides exacerbate this permeability, while activated coagulation pathways may cause microvascular obstructions, further impairing endothelial integrity. These combined effects allow fluids and proteins to leak from the vascular space into the myocardial interstitium, leading to myocardial edema. This edema restricts the expansion of cardiac chambers and impairs contractility, thus decreasing the efficiency of the heart muscle.

The decreased plasma colloid osmotic pressure is another mechanism contributing to increased fluid filtration rate. Experimental data in rabbit hearts support this assertion, showing increased microvascular permeability associated with reduced circulating protein levels(albumin) [88]. (Fig. 2). Similar phenomena are observed in working rat models, where ME is successfully induced by crystalloid coronary perfusion and resolves within 5 min of blood reperfusion [89]. In pig heart models, crystalloid coronary perfusion has also been shown to produce ME and diastolic stiffness, which resolve after 45 min of blood reperfusion, showing that a similar mechanism may apply to larger animal models [90,91]. Although similar experiments have not been reported in humans, the lack of plasma colloids in cardioplegic solutions could impair myocardial microvascular permeability, as well-documented post-operative ME shows in patients undergoing cardiopulmonary bypass [84]. Since it is a known fact that inflammation increases capillary permeability and allows filtration of serum albumin [92], gradual systemic hemodilution from albumin depletion in the context of inflammation is expected to contribute to the formation of ME. Therefore, maintaining a certain circulating protein (albumin) is necessary to preserve normal microvascular permeability.

#### 2.5.2. Implication of Bradykinin Receptor B1 (B1R) in myocardial edema

Recently, the role of kinin receptors in sepsis has become an exciting focus of study. Kinins are proteins released in injured tissues from kininogens either by tissue kallikreins or plasma kallikreins in sites of inflammation [93,94]. They are responsible for a variety of vascular responses (smooth muscle contraction/relaxation, vasodilatation, increased vascular permeability, and pain) through their interaction with two G protein-coupled kinin receptor subtypes: the kinin B2 receptor (B2R) and the kinin B1 receptor (B1R) [94]. B2R is constitutively expressed under physiological conditions in different cell types and is believed to mediate the acute inflammatory response after a harmful stimulus [94]. As opposed to B2R, B1R is normally under-expressed and is inducible in the presence of certain pro-inflammatory cytokines, thus responsible for the sustained inflammatory response [94]. On endothelial cells, activation of B2R generates small amounts of NO through activation of eNOS and Akt activation and phosphorylation of Ser1177. However, in endothelial cells under inflammatory conditions, B1R stimulation results in a higher and prolonged NO production via Gαi, Gβγ, and Src-dependent activation of the ERK/MAP kinase pathway leading to activation of iNOS via phosphorylation at Ser745 [95]. B1R is upregulated in inflammatory conditions, and its activation can lead to increased vascular permeability and contribute to the pathophysiology of sepsis. Moreover, the expression of B1R is believed to destabilize Vascular Endothelial cadherin (VEcadherin), an adherens junction protein, which impairs the endothelial barrier function and increases microvascular permeability [96,97]. This is supported by a recent in vivo study in a polymicrobial rodent sepsis model, in which B1R blockade helped reduce VE-cadherin disruption by limiting eNOS activation, consequently maintaining a higher MAP and resulting in higher survival rates in the treated group [98]. There are ongoing investigations into using B1R inhibitors to address endothelial permeability, particularly in sepsis. Bradykinin receptors, specifically B1R, play a role in vascular leakage and inflammation during sepsis [18,19,99]. As the link between B1R and vascular permeability is established, B1R could also act as a determinant factor in forming ME, which may contribute to myocardial depression in sepsis. However, the use of B1R blockade specifically in treating SICM is underexplored, and further clinical trials are needed to confirm their efficacy and safety in human subjects.

#### 2.5.3. Glycocalyx alterations in Sepsis ME: A new diagnostic and therapeutic target

The increase in microvascular permeability is also secondary to disrupted endothelial function, contributing to ME formation. This is believed to be the consequence of many factors, including decreased endothelial tight junctions and increased leukocyte adhesion secondary to upregulated cell adhesion molecules in the context of inflammatory stimuli [82]. ECM-degrading activity has also been observed in post-mortem SICM samples, presumably secondary to high levels of ECM-degrading enzymes expressed by interstitial macrophages (Fig. 2). Additionally, sheddases, such as metalloproteinases, heparinase, and hyaluronidase, are activated by ROS and inflammatory cytokines in sepsis [100] to subsequently damage the glycocalyx, which is a negatively charged layer of proteoglycans, glycosaminoglycans, and absorbed plasma proteins coating the luminal surface of the microvascular exchange vessel endothelium [101] (Figure 2). The disruption of the glycocalyx has been reported to alter microvascular permeability [102]. In sepsis-induced rat models, negative charges have decreased in the glycocalyx and the basement membrane of myocardial capillary endothelial cells [103]. With increased myocardial microvascular permeability, plasma colloids leak into the myocardial interstitium and draw fluid along with them, thus leading to the formation of ME.

The important role of glycocalyx disruption in microvascular barrier dysfunction during sepsis suggests that glycocalyx disruption may become an interesting diagnostic and therapeutic target in SICM. Circulating levels of syndecan 1, a core protein of the glycocalyx, are observed to increase in greater quantities in septic patients when compared to surgical patients, thus making it a possible biomarker to detect extensive glycocalyx damage in SICM [104,105]. Circulating endocan levels, another component of the glycocalyx, can be measured following its release in response to TNF-α and IL-1 during sepsis. Since endocan is observed to correlate with the severity of sepsis [106,107], it may become a possible biomarker to evaluate glycocalyx degradation in SICM. Despite the significant role of glycocalyx disruption in sepsis, targeted therapeutic options remain under exploration. Anticoagulant molecules, such as antithrombin or activated protein C, are theorized to preserve glycocalyx integrity [108] but failed to improve patient outcomes in septic shock [109]. A vascular leakage blocker, CU06–1004, is believed to improve cardiac function by inhibiting cardiac microvascular endothelial cell hyperpermeability and reducing the neutrophil’s plugging and infiltration in infarcted hearts [110]. However, the blocker has yet to be tested in septic animal models, and whether inhibition of the vascular permeability could prevent SICM is unknown.

#### 2.5.4. Microvascular dynamics in ME: Balance of filtration and lymphatic drainage

Like other organs, lymphatic outflow is the primary mechanism for removing interstitial edema from the myocardial interstitium. Particularly in the heart, the lymphatic vessels are valveless and depend solely on myocardial contractions for fluid propulsion [111]. In SICM, the initial ME disrupts the normal anatomy of the myocardial lymphatic network, which reduces lymphatic drainage, as supported by the observation of lymphatic collapse in post-mortem samples [82]. This added up to the reduced cardiac contractility observed in SICM, which compromises lymphatic outflow, creates a vicious cycle that favors the accumulation of interstitial fluid (ME) in the septic heart (Fig. 2). This mechanism partially explains the heart’s sensitivity to slight volume increases in the myocardium.

#### 2.5.5. Cardiac function sensitivity to slight volume increases in the myocardium

ME formation has been shown to substantially decrease global cardiac function, which does not immediately improve upon the resolution of ME [112]. As mentioned before, the heart is extremely sensitive to increased interstitial volume, and it has been reported that an increase of 3.5% of myocardial water content secondary to coronary sinus pressure elevation decreases cardiac output by 40% for a given pre-load volume [113]. This is primarily believed to be a consequence of increased ventricular chamber stiffness, supported by studies correlating increased ventricular diastolic stiffness with ME formation [114–116]. The altered viscoelastic properties of the myocardium can compromise ventricular chamber compliance, increasing the diastolic filling pressure and wall tension, simultaneously impairing microvascular flow and aggravating myocardial injury [117]. This is supported by in vivo rat and guinea pig models in which the accumulation of ME is associated with increased cardiac energy consumption [118]. The mechanism behind these phenomena could be explained by a disruption of collagen fiber anchoring points secondary to increased interstitial volume and pressure during ME, which alters the normal collagen structure upon which the heart normally functions, consequently impacting cardiac function as observed in animal models with collagen abnormality [119,120]. The accumulating edema also increases the diffusion distance for oxygen to reach cardiomyocytes, resulting in great functional impairment since the heart always operates on near maximum oxygen extraction capacity (Fig. 2). This agrees with data demonstrating increased coronary vascular resistance associated with ME in rat ME models [121].

## 3. Current treatment options for SICM and future directions

Despite advancements in medical treatments, sepsis-induced cardiomyopathy (SICM) continues to exhibit high mortality and morbidity rates. Understanding SIMD’s mechanisms and potential therapeutic strategies is essential for improving treatment and reducing mortality. SIMD displays heterogeneity, including the severity of sepsis, preexisting cardiac conditions, and the patient’s overall health [29]. SIMD progresses through different stages. In the early hyperdynamic phase, patients can exhibit elevated cardiac output, reduced systemic vascular resistance, and tachycardia. This compensatory response helps to maintain perfusion despite systemic inflammation. As sepsis progresses, continued inflammation and direct myocardial injury exacerbate endothelial damage, further increasing permeability and myocardial edema. This transition to the intermediate and late stages, characterized by myocardial depression and reduced ejection fraction, often leads to hypotension and shock, which is often associated with poor prognosis and high mortality. Diagnostic tools like echocardiography and cardiac MRI are crucial for detecting these changes early [122,123]. Echocardiography can initially reveal increased myocardial wall thickness and hyperdynamic function, while later stages show impaired contractility [124]. Cardiac MRI provides detailed visualization of myocardial edema and can help adjust therapeutic interventions [32,125]. Early fluid management and anti-inflammatory treatments are critical to reducing edema, while later stages may require inotropic support and vasopressors to maintain cardiac function. Understanding and addressing the variability in cardiac response through advanced imaging and targeted therapies can significantly improve outcomes for patients with SIMD.

## 4. Limitations and challenges of current therapies for SICM

Current treatments for septic-induced cardiomyopathy (SICM) mainly focus on inflammation suppression and hemodynamic stabilization but often prove inadequate, which might be due to several critical factors. Firstly, the timing of intervention is crucial; the effectiveness of anti-inflammatory treatments is highly dependent on the timing of administration. Delayed intervention, after the cytokine storm has been initiated, may result in limited efficacy [126]; Furthermore, targeting specific cytokines in sepsis-induced heart failure treatment is challenging due to their redundancy and pleiotropy. This complexity can reduce the effectiveness of treatments that focus on a single cytokine, indicating the need for therapeutic approaches that can disrupt multiple pathways [127]. The cytokine storm not only exacerbates cardiac injury but also reduces the efficacy of conventional therapies to stabilize cardiac function. Importantly, current therapeutic strategies do not directly control the dysregulated immune response.

### 4.1. Clinical available treatment for SICM

Fluid resuscitation and vasopressors are considered first-line treatments to stabilize hemodynamic instability, often due to vasodilation and increased vascular permeability [128]. The debate between using crystalloid and colloid solutions as first-line fluid resuscitation has been ongoing for decades and remains unsolved. Current guidelines preferer crystalloids (due to lower cost) over colloids, as there is a lack of clinical evidence suggesting a difference in patient outcomes with colloids, specifically albumin [129]. Despite its theoretical advantage in maintaining plasma oncotic pressure, robust studies did not find any difference in mortality when comparing albumin to crystalloids [129,130]. Crystalloids have been associated with lower static filling pressures and mean arterial pressure (MAP) in critically ill patients [131]. Despite the lack of data, the use of albumin to prevent hemodilution in patients who have received large amounts of crystalloids is supported by evidence showing higher blood pressure at early and later time points [129], higher static filling pressures [131], and lower net fluid balance [129]. The use of gelatin, a synthetic colloid, has been introduced recently, though there is also insufficient data to justify its superiority or inferiority to crystalloids.

Vasoactive agents are used when fluid alone is insufficient to reach MAP targets. Current guidelines recommend norepinephrine as a first-line vasopressor due to its ability to effectively increase blood pressure with minimal effects on heart rate, which is crucial in managing the hemodynamic instability seen in sepsis. Vasopressin is recommended as a second-line therapy if needed [128].

In cases of SICM, often suspected in patients with low cardiac output (CO) and elevated cardiac filling pressures that do not respond effectively to fluids and vasopressors, inotropic therapy can be used. The most commonly used inotropes are dobutamine and epinephrine [128]. Levosimendan has been added as an inotropic agent alternative in recent years, but no difference in mortality has been noted in studies so far when compared to dobutamine [128]. Nevertheless, long-term effects in survivors of septic shock, such as cardiac edema preservation, would be an interesting avenue for studies comparing crystalloid to colloid re-suscitations, potentially providing valuable insights into preventing and treating SICM. Recent literature has also suggested that side effects from necessary therapeutic interventions, notably antibiotics, may disturb the intestinal microbiome diversity and contribute to the dysregulated inflammatory response in sepsis, likely from the loss of health-promoting short-chain fatty acids such as butyrate [132]. While fecal microbiota transplant (FMT) has shown promise in recovery, this intervention and its availability prevent its use as a standard therapeutical option [132].

### 4.2. Potential therapeutic targets and future directions

In addition to current treatments, several promising therapeutic targets for sepsis-induced cardiomyopathy (SICM) focus on reducing inflammation, including Bradykinin Receptor B1 (B1R) antagonists [18], Toll-Like Receptor (TLR) modulation, NF-κB inhibition [133], Formyl Peptide Receptor 2 (FPR2) agonists such as Annexin A1 (AnxA1) [134], and pro-resolving lipid mediators like resolvins and lipoxins, along with Interleukin-1 Receptor Antagonist (IL-1Ra), which show potential in improving cardiac outcomes [135]. Future research may prioritize developing therapies that specifically target and reduce cardiac edema at the earliest stages of increased permeability. Including targeting B1R to reduce myocardial microvascular permeability, stabilizing endothelial junctional proteins to maintain vascular integrity, and modulating mitochondrial function to decrease endothelial permeability. Early intervention with anti-edema therapies and integrating insights from immunology and cardiology to attenuate immune response-induced edema without compromising infection-fighting capabilities are also crucial. By focusing on these strategies, we can pave the way for innovative treatments that offer hope for improved outcomes in SICM patients.

## 5. Conclusion

Despite the absence of a universally accepted definition, SICM is increasingly recognized as a critical factor contributing to hemodynamic instability and poorer outcomes in septic shock patients. Over the past decade, significant advances have been made in understanding the pathophysiology of SICM, shifting focus from solely inflammatory cytokines to a broader exploration of excessive nitric oxide production, mitochondrial dysfunction, calcium homeostasis disturbance, and notably myocardial edema. This evolving understanding is key to developing more effective treatments. As sepsis continues to be a leading cause of mortality globally, further research into these mechanisms is crucial for enhancing patient survival rates worldwide.
